# BRONCO: Biomedical entity Relation ONcology COrpus for extracting gene-variant-disease-drug relations

**DOI:** 10.1093/database/baw043

**Published:** 2016-04-13

**Authors:** Kyubum Lee, Sunwon Lee, Sungjoon Park, Sunkyu Kim, Suhkyung Kim, Kwanghun Choi, Aik Choon Tan, Jaewoo Kang

**Affiliations:** 1 Department of Computer Science and Engineering, Korea University, 145 Anam–ro, Seongbuk–gu, Seoul, 02841 Korea and; 2 Translational Bioinformatics and Cancer Systems Biology Laboratory, Division of Medical Oncology, Department of Medicine, University of Colorado Anschutz Medical Campus, 12801 East 17th Avenue Aurora, CO 80045, USA

## Abstract

Comprehensive knowledge of genomic variants in a biological context is key for precision medicine. As next-generation sequencing technologies improve, the amount of literature containing genomic variant data, such as new functions or related phenotypes, rapidly increases. Because numerous articles are published every day, it is almost impossible to manually curate all the variant information from the literature. Many researchers focus on creating an improved automated biomedical natural language processing (BioNLP) method that extracts useful variants and their functional information from the literature. However, there is no gold-standard data set that contains texts annotated with variants and their related functions. To overcome these limitations, we introduce a Biomedical entity Relation ONcology COrpus (BRONCO) that contains more than 400 variants and their relations with genes, diseases, drugs and cell lines in the context of cancer and anti-tumor drug screening research. The variants and their relations were manually extracted from 108 full-text articles. BRONCO can be utilized to evaluate and train new methods used for extracting biomedical entity relations from full-text publications, and thus be a valuable resource to the biomedical text mining research community. Using BRONCO, we quantitatively and qualitatively evaluated the performance of three state-of-the-art BioNLP methods. We also identified their shortcomings, and suggested remedies for each method. We implemented post-processing modules for the three BioNLP methods, which improved their performance.

**Database URL**: http://infos.korea.ac.kr/bronco

## Introduction

Modern next-generation sequencing (NGS) technologies have revolutionized modern biomedical research. In particular, cancer genomics studies that use NGS have identified novel somatic alterations such as single-nucleotide variants, insertions and deletions, copy number aberrations, structural variants and gene fusions as actionable targets in cancer. Variant annotation is a key step in the analysis of cancer genomics data. Many thousands of cancer genomes and exomes have been sequenced; however, the efforts in variant annotation have not been able to keep up with the identified variants. Large-scale cancer genome projects such as The Cancer Genome Atlas (1) and the International Cancer Genome Consortium (2) found the following two key findings: (i) the alterations in cancer genome are complex and (ii) every cancer genome is different. Information on variants correlated with drug response can help clinicians tailor treatment for individual patients. However, the functional annotation of variants can profoundly impact the conclusions of disease studies. For example, incorrect or incomplete annotations could cause researchers to overlook disease-relevant variants or label interesting variants as false positives.

Many novel computational tools and methods focus more on identifying variants from NGS data than on annotating them. Existing resources and tools can be classified into three categories: (i) prediction-based such as SIFT (3) and PolyPhen (4); (ii) reference-based such as dbSNP (5), 1000 Genomes Project (1KG) (6), NHLBI ESP and ENCODE (7); and (iii) curation of variants in the literature using biomedical natural language processing tools (BioNLP tools). Manual curation approaches usually produce higher quality results, and present variants in the context of biology. Experts manually extract variants from published literature and provide biological annotation in a database (Supplementary Table S1) such as My Cancer Genome (8). However, these approaches are laborious, and annotate only a small number of variants. Moreover, various groups of experts use different standards for annotating variants.

Recognizing that it is impossible to manually curate all variants from the literature, some experts have integrated BioNLP approaches for processing annotations (Supplementary Table S2). BioNLP systems use computational tools and methods that perform information retrieval, document classification, information extraction or literature-based discovery of human-generated texts (e.g. abstracts, full-text articles, patents). Some knowledge bases such as PharmGKB (9) were developed using BioNLP approaches (10).

However, several challenges exist in using BioNLP methods for variant extraction and annotation. Extracting variants using BioNLP systems is a difficult task because there may be different forms of the same mutation in the literature. To address this challenge, the Human Genome Variation Society (HGVS) (http://www.hgvs.org/mutnomen/) has made recommendations for nomenclature of variations (11); however, many researchers and published papers do not follow the recommendations, which makes it even more difficult for BioNLP systems to extract variants. Supplementary Figure S1 shows examples of variations that use HGVS nomenclature.

The lack of comprehensive and well-annotated full-text corpora for training and evaluating computational methods represents another limitation of the BioNLP systems. Previous studies have performed evaluation using abstract-only corpora (12, 13), corpora that are biased toward single nucleotide variants (14), or corpora (e.g. Variome corpus) (15, 16) that contain only a limited number of full-text articles. Moreover, most of the existing annotated corpora contain variants without any biological context (12, 14).

To overcome these limitations, we developed BRONCO—a new Biomedical entity Relation ONcology COrpus. BRONCO contains >400 variants and their relations with genes, diseases, drugs and cell lines in the context of cancer, all of which were extracted from 108 full-text articles. These relations (e.g. variant-gene, variant-disease, variant-drug, variant-cell line) have not been comprehensively annotated in existing corpora. We believe that these biological relations can be useful to next-generation BioNLP systems in extracting variants within a biological context. Furthermore, we performed a systematic cross-validation of several state-of-the-art BioNLP systems using BRONCO. We identified several weaknesses of each tool, developed a post-processing module to refine the existing methods’ results, and provided a standardized corpus for objectively evaluating BioNLP systems.

The structure of this paper is as follows. First, we describe the construction of BRONCO. Next, we quantitatively and qualitatively evaluate MutationFinder (MF), extractor of mutation (EMU) and tmVar, all of which are state-of-the-art variant extraction methods, using three public data sets and BRONCO. Last, we outline the limitations of the current BioNLP systems and the areas that can be improved in future work.

## Methods

### Biomedical entity Relation ONcology COrpus

*Literature collection of BRONCO*. We developed BRONCO—a Biomedical entity Relation ONcology COrpus—which is a variant-centric data set with related genes, diseases, drugs and cell lines. To collect free full-text articles from PubMed Central (PMC) for the corpus, we used Medical Subject Headings (MeSH). We used the following query in PMC: ‘cell line’ [MeSH Terms] AND ‘mutation’ [MeSH Terms] AND ‘drug screening assays, antitumor’ [MeSH Terms]. We retrieved about 200 free full-text papers published between August 2009 and August 2014. We used MF, EMU and tmVar to find papers that contain mutations. We used BioNLP in this step to classify the 200 papers into Group A and Group B. Papers that contained more than two variants were classified under Group A, and papers that contained two or less variants were categorized under Group B. The 108 papers in Group A were manually curated for BRONCO. We have manually reviewed all the 100 papers in Group B, and confirmed that none of these papers contained more than two variants (a criterion that we used to determine whether a paper should be included in BRONCO). To be precise, 6 papers contained two variants, 16 papers contained only one variant and the remaining papers did not contain any specific variants that fulfilled our annotation guidelines.

**Figure 1.**
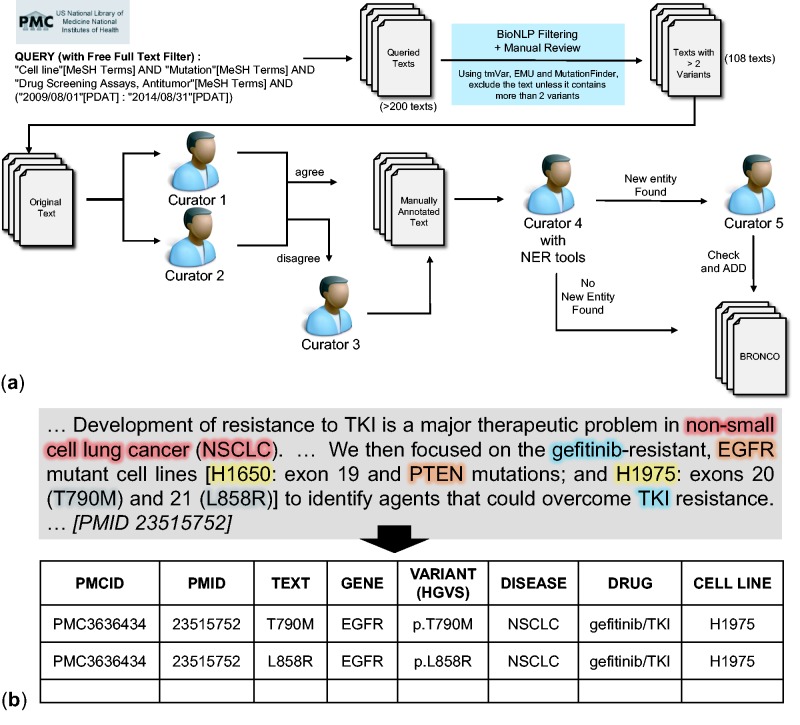
Manual curation of BRONCO. (a) Workflow of manual curation. (b) Example of manual curation.

*Manual annotation of BRONCO*. A total of 108 full-text articles in BRONCO were manually curated by biomedical experts. We followed HGVS nomenclature when curating BRONCO. We included not only the variant mentions that were in wild-type residue + amino acid position + mutant residue (wNm) form (e.g. T790M, Thr790Met) but also the variant mentions in simple natural language form (e.g. threonine 790 substituted with methionine), and dbSNP form (e.g. rs121434569). We excluded some variants with insufficient information (e.g. valine 600 is mutated). When variant mentions were found, the variant-related entities such as genes, diseases, drugs and cell lines were curated. Figure 1 illustrates the curation workflow of BRONCO. At least two manual curators were assigned to one article and instructed to manually curate variants and related entities (genes, diseases, drugs and cell lines). Additional manual curators decided cases on which expert curators disagreed. To check for missing information, another curator compared the manually curated results with the results that were curated using BioNLP tools. The cases that were found using BioNLP tools [tmVar, MF, EMU and BEST Biomedical Entity Extractor (BEST Biomedical Entity Extractor: http://infos.korea.ac.kr/bioentityextractor/) (17)] were double checked by another curator if they were likely to be true positive but were excluded in a previous curation. This process is illustrated in Figure 1a. The curators numbered 1–5 are all different. If needed, four manual curators and one semi-automated curator may be used. All our curators have a graduate degree in biomedical sciences. The manual curation example is shown in Figure 1b and the guidelines that were provided to the manual curators are attached as a Supplementary file. BRONCO is freely available at http://infos.korea.ac.kr/bronco.

### Published corpora

*Published corpora.* We downloaded the gold-standard data sets that were developed to evaluate the performance of mutation extraction systems. The data sets that were used for testing MF, EMU and tmVar contain 508, 502 and 500 abstracts, respectively. These data sets come with their own manually curated answer sets. Each answer set was presented in different formats. The answer set of the EMU data set is available for the 51 prostate cancer and 121 breast cancer related mutations extracted from a total of 109 abstracts. Out of 502 abstracts in the EMU data set, we used the 109 abstracts that contained mutations.

*Unified format for answer sets*. All the data sets and the result files of algorithms have their own file format, different data fields and different standards for manual curation. For example, the MF corpus contains only document IDs and lists of variants in wNm form whereas the tmVar corpus contains texts of original documents, and the EMU corpus contains related gene information. The differences in the file formats and curation standards of each corpus make it difficult to objectively evaluate and compare the corpora. For example, we ran EMU and tmVar on the MF data set and found that many of the false positives in the result were reclassified as true positives. These variants were false positives because they were originally excluded from the MF answer set. Many variants are also excluded from EMU and tmVar answer sets because of the differences in the guidelines of manual curation. We went through all the false positives and false negatives that were identified using each method, and manually corrected the answer sets. To facilitate the comparison of different data sets, we developed a unified file format for all answer sets. This format contains all the information in each data set, which can be used for future use. All the updated answer sets in unified format are available at http://infos.korea.ac.kr/bronco.

### BioNLP methods

In this study, using BRONCO and three other published corpora, we evaluate the following three BioNLP methods: MF, EMU and tmVar. We also developed two ensemble approaches: simple merging (SM) and majority voting (MV).

*MutationFinder*. MF (14) was developed to extract point mutations from the literature using a rule-based approach. It focuses on extracting point mutations in wild-type residue + amino acid position + mutant residue (wNm) form (e.g. T790M, Thr790Met) or in simple natural language form (e.g. threonine 790 substituted with methionine). It uses a set of regular expressions for extracting these mutations. MF was downloaded from http://mutationfinder.sourceforge.net.

*Extractor of mutations*. EMU is another commonly used variant extraction method (13). It extracts not only point mutations but also insertion/deletion mutations. EMU uses more regular expressions for extracting mutations in various forms including complex natural language variant forms. EMU also extracts related gene information of each mutation using the co-occurrence of genes and mutations in a text. EMU uses a ‘fallible list’ that contains cell-line names in wNm form to filter false positive mutations. EMU also provides an additional online gene filtering tool that finds matching genes for each variant using gene sequence information. When the EMU module extracts a variant from a text, it also extracts a list of candidate genes in the same text. The filtering tool finds the amino acid sequence of the genes from the NCBI RefSeq (http://www.ncbi.nlm.nih.gov/refseq/) online API and compares it with the wild-type amino acid of the variant. If the amino acid sequence does not match the wild-type of the variant, the gene names in the gene list are discarded. The result obtained using EMU contains mutation types, related genes and the location of variants in the document. EMU was downloaded from http://bioinf.umbc.edu/EMU/ftp.

*tmVar*. tmVar is a text-mining approach that is based on a conditional random fields (CRF) and used for extracting a wide range of variants (point mutations, insertions/deletions, frameshifts) described at protein, DNA and RNA levels (12). To identify mutations accurately, tmVar uses six different features (including linguistic and semantic features) for the CRF algorithm. In addition, tmVar incorporates regular expressions as a post-processing step. The two-step approach of tmVar was reported to obtain high precision and recall rates. tmVar was downloaded from http://www.ncbi.nlm.nih.gov/CBBresearch/Lu/pub/tmVar/.

*Majority voting*. We used the three BioNLP methods as the base extractors in MV. The final assignment of a variant is based on two of the three methods that extracted the same variant.

*Simple merging*. In contrast to the MV approach, the SM approach assigns variants extracted by at least one of the methods.

### Assessment methods

*Assessment methods*. We used precision, recall and F1-score to evaluate the BioNLP methods’ performance on different data sets. Precision is a true positive number divided by the total number of results decided as true by a method, and represents the purity of the results retrieved using the method. Recall is the true positive number divided by the number of true mutations in the literature, and represents the coverage and the variety of mutation forms that each method can retrieve. F1-score is the harmonic mean of precision and recall.

$$\begin{array}{l} \text{Precision}=\left(\text{True Positive}\right)/\left(\text{True Positive}+\text{False Positive}\right)\\ \text{Recall=}\left(\text{True Positive}\right)/\left(\text{True Positive}+\text{False Negative}\right)\\ \text{F1-score }=\;\left(2\;\times \text{ Precision }\times \text{ Recall}\right)/\left(\text{Precision}+\text{Recall}\right) \end{array}$$

**Figure 2.**
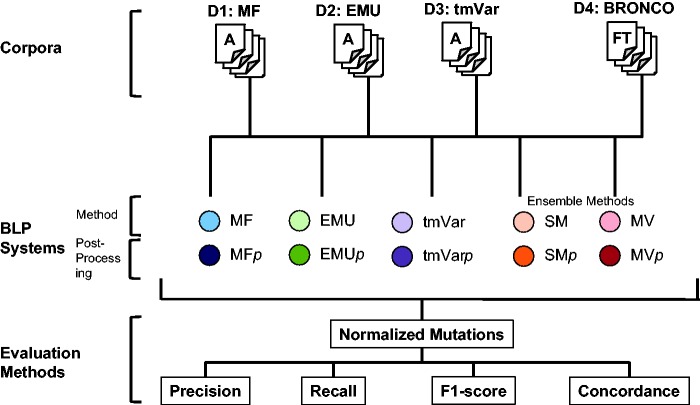
Workflow for assessing the performance of MF, EMU and tmVar in this study.

The following three metrics can be used in deciding whether results are correct: Extracted Mentions, Normalized Mutations and Document Retrieval (14). Each mention of a variant must be identified when calculating Extracted Mentions. This metric counts all the mentions of a mutation even though the mutation appears several times. For example, if the T790M mutation appears in a document twice, the method can obtain a perfect score if it extracts T790M twice when calculating Extracted Mentions. The Normalized Mutations metric counts the same mutation as one entity. In the same example, when calculating Normalized Mutations, a method can obtain a perfect score if it extracts the mutation in the document at least once. Document Retrieval checks whether a document contains any mutations in a binary way but does not consider the type of a mutation or the number of times it appears in a document. In this study, we used Normalized Mutations to evaluate the BioNLP methods as it is provided as the ‘answer set’ for each published data set. The overall workflow of this study is illustrated in Figure 2.

## Results

### Biomedical entity Relation ONcology COrpus

*BRONCO overview*. BRONCO is currently the largest full-text variant-centric corpus annotated with related genes, diseases, drugs and cell-line information. BRONCO contains 108 full-text articles, whereas other corpora from previous studies contain only abstracts. Table 1 lists the characteristics of BRONCO and those of the other four corpora. BRONCO focuses on papers published in the cancer research domain as there are more mutations reported in them. We attached the guidelines for the manual curation and the curation example file that we provided to the curators as supplementary files.

**Table 1.** Characteristics of the variant-centric corpora

Corpus	MF	EMU	EMU (New)	tmVar	Variome	BRONCO
Type	Title and abstract	Title and abstract	Title and abstract	Title and abstract	Full-text	Full-text
Contained Information	Variants	Variants-genes-diseases (BC, PC)	Variants-genes-diseases (BC, PC)	Variants	Annotated Texts	Var-gene-disease-drug-cell-line
Total number of docs	508	109	109	500	10	108
Word count	107 812	25 495	25 495	119 649	42 921	505 311
File size (kbytes)	714	169	169	806	272	3405
Normalized mutation	482	179	287	1057	52	403
Extracted mutation	Unknown	Unknown	Unknown	1410	118	2311
Publication period	Nov. 1969–Feb. 2006	1994–May. 2008	1994–May. 2008	2002–Apr. 2012	Oct. 2005–Jan. 2011	Sep. 2009–Apr. 2014
Mutation Types	Sub	Sub	Sub, Del, Ins, SNP, FS	Sub, Del, Ins, Dup, InDel, SNP, FS	Sub, Del, Ins, FS	Sub, Del, Ins, InDel, SNP, FS
Unique Var	451	172	237	871	50	275
Var with genes		179 (100%)	179 (75.53%)		(Unknown)	403 (100%)
Var with diseases		170	170		(Unknown)	338 (83.87%)
Var with drugs					(Unknown)	202 (50.12%)
Var with cell lines					(Unknown)	182 (45.16%)

*Statistics of BRONCO.* In the manual annotation step, all 108 papers were manually curated at least twice and out of all the 108 papers, 27 papers were manually curated multiple times because the annotators had a difference of opinion. The most unique feature of BRONCO is that it contains 403 variants and their relations with genes, diseases, drugs and cell lines. As the collected papers focused on cancer research, many of the variants extracted from the papers were related to oncogenes or tumor-suppressor genes listed in the Cancer Gene Census (COSMIC) database (The COSMIC: http://cancer.sanger.ac.uk/census) (18). 83.87% of the variants were mentioned with related diseases. Out of all the 358 disease-related variants, 341 variants were related to tumors, and other diseases/symptoms such as skin lesions, thrombocytosis, myelofibrosis and hematopoiesis were indirectly related to tumors. 50.12% and 45.16% of the variants were mentioned with drugs and cell lines, respectively. There are 66 unique genes, 64 diseases, 133 drugs and 154 cell lines in BRONCO. Out of the 403 variants in BRONCO, there are 385 substitutions (95.5%), 9 deletions (2.2%), 2 insertions, 6 frameshifts and 1 Single-nucleotide polymorphism (SNP) with a dbSNP identifier.

*Comparison with other corpora.* Other public corpora do not contain rich information about variants such as relations with other biomedical entities. The EMU corpus contains only gene and disease (only breast cancer and prostate cancer) related information of each variant. The Variome corpus (16) contains gene and disease tagging information of 10 full texts; however, since the Variome data set is a list of text tagging information, it is difficult to know the relations between diseases, genes and variants. The corpora of MF and EMU contain only point mutation information whereas other corpora contain information about various mutation types. For the objective evaluation, we found additional mutations such as deletions, insertions, frameshifts and SNPs using semi-manual curation and added them to the MF corpus and the EMU corpus.

### Comparison of the performance of different BioNLP methods

We first compared the three BioNLP methods’ performance on the three publicly available data sets, using the unified format answer sets for assessing precision, recall and F1-score (Figure 2). Using the same data, we also compared the results obtained using SM and MV. Table 2 lists the results of these comparisons. Supplementary Figure S3 illustrates all the results in a Venn diagram.

**Table 2.** Precision, recall and F1-score of the methods tested on four corpora.

Method	Measure	Corpora												Average		
		MF			EMU			tmVar			BRONCO					
		Orig.	P.P.	Imprv. (%)	Orig.	P.P.	Imprv. (%)	Orig.	P.P.	Imprv. (%)	Orig.	P.P.	Imprv. (%)	Orig.	P.P.	Imprv. (%)
MutationFinder	Precision	0.985	0.985	0.00	0.995	0.995	0.00	0.985	0.985	0.00	**0.915**	0.934	2.08	**0.970**	0.974	0.41
	Recall	0.805	0.801	−0.50	0.747	0.743	−0.54	0.294	0.292	−0.68	0.876	0.876	0.00	0.681	0.678	−0.44
	F1-score	0.886	0.883	−0.34	0.854	0.851	−0.35	0.453	0.451	−0.44	**0.895**	0.904	1.01	0.772	0.772	0.00
EMU	Precision	0.977	0.982	0.51	0.956	0.973	1.78	0.845	0.932	**10.30**	0.773	0.847	9.57	0.888	0.934	5.18
	Recall	0.801	0.797	−0.50	0.959	0.955	−0.42	0.699	0.711	1.72	0.903	0.906	**0.33**	0.841	0.842	**0.12**
	F1-score	0.880	0.880	0.00	0.957	0.964	0.73	0.765	0.807	**5.49**	0.833	0.876	5.16	0.859	0.882	2.68
tmVar	Precision	0.985	0.988	0.30	0.988	0.988	0.00	0.955	0.972	1.78	0.767	0.881	14.86	0.924	0.957	3.57
	Recall	0.842	0.838	−0.48	0.952	0.944	−0.84	0.937	0.930	−0.75	0.938	0.933	−0.53	0.917	0.911	−0.65
	F1-score	**0.908**	**0.907**	−0.11	0.970	0.966	−0.41	**0.946**	**0.951**	0.53	0.844	0.906	7.35	**0.917**	**0.932**	1.64
Simple Merging	Precision	0.956	0.962	**0.63**	0.950	0.967	**1.79**	0.852	0.930	9.15	0.684	0.795	**16.23**	0.861	0.914	**6.16**
	Recall	**0.851**	**0.846**	−0.59	**0.996**	**0.989**	−0.70	**0.948**	**0.942**	−0.63	**0.943**	**0.941**	−0.21	**0.934**	**0.930**	−0.43
	F1-score	0.900	0.901	**0.11**	**0.973**	**0.978**	**0.51**	0.897	0.936	4.35	0.792	0.862	**8.84**	0.891	0.919	**3.14**
Majority voting	Precision	**0.995**	**0.995**	0.00	**0.996**	**0.996**	0.00	**0.989**	**0.992**	0.30	0.867	**0.941**	8.54	0.962	**0.981**	1.98
	Recall	0.809	0.803	−0.74	0.926	0.918	−0.86	0.694	0.708	**2.02**	0.906	0.906	0.00	0.834	0.834	0.00
	F1-score	0.892	0.889	−0.34	0.960	0.956	−0.42	0.816	0.826	1.23	0.886	**0.923**	4.18	0.889	0.898	1.01

The highest scores are highlighted in bold in each corpus. Orig, original results; P.P., post-processing results; Imprv., improvement of the post-processing results over original results.

As the results show, MF, a simple regular expressions based method used for extracting variants, has the highest precision and the lowest recall among the five methods. MF achieved the lowest recall on the tmVar corpus, which is expected as it identified only point mutations in wNm form and limited the number of mentioned variants in natural language form. The tmVar corpus contains other mutations (e.g. insertions, deletions, frameshifts) that cannot be extracted using MF. The MV and tmVar approaches had the second and third highest precision, respectively.

From this study, SM achieved the highest recall on these four corpora. SM will assign a variant as long as one of the three base methods identifies it in a text. Out of the three BioNLP methods, tmVar can find the most various forms of variants and obtain the best recall on the tmVar corpus.

As shown in Table 2, tmVar achieved the highest F1-score. This is interesting as tmVar consistently achieved high precision and high recall on all the corpora in this study. SM and MV had the second and third highest F1-scores on average, respectively.

As BRONCO consists of full-text articles that are objectively selected by a keyword search, it reflects more closely the actual distribution of the different types of mutations mentioned in the literature. In BRONCO, mutations in wNm form, which adhere to the HGVS annotation format, are more frequent than mutations in other forms. Therefore, it is not surprising that MF, a first-generation BioNLP method that employs simple rules, achieved high precision and F1-score on BRONCO.

Based on our assessment using BRONCO, tmVar and EMU have lower precision than MF. tmVar and EMU extract many mutation-like texts that are in Alphabet-Number-Alphabet form, which achieves good recall but relatively lower precision on the evaluation results. When tmVar and EMU are used to extract variants from full texts, compared with abstracts, there are more texts in Alphabet-Number-Alphabet form that are not variants. Some of the false-positive results obtained using tmVar and EMU on the BRONCO data set were figure numbers, chemical formulae or other biomedical entities. MF extracts variants only when texts in Alphabet-Number-Alphabet form do not contain any symbols or spaces; however, tmVar extracts variants even when there are symbols or spaces in the form, and achieves higher recall but lower precision than MF.

However, these three tools are designed and trained using abstract texts. As Cohen *et al.* (19) explained, the abstract and the body of a full-text article have different properties. If the tools are re-designed and re-trained using BRONCO, they will achieve better results. Also, this reminds us again why a full-text variant corpus such as BRONCO is needed for building better tools.

### Post-processing module for improving variant detection

Next, we asked whether these methods of extracting mutations from text could be further improved. We carefully analysed the results obtained by each method, and found that there were some consistent false positives identified by each method. Many of these false positives can be easily fixed. Thus, we have developed a post-processing module that improves the performance of all the methods. This post-processing module takes the output files or the unified forms of each BioNLP system, which are mentioned in the Methods section, as input. This post-processing module is publically available at http://infos.korea.ac.kr/bronco.

In brief, the post-processing module incorporates the following rules:

1. Filtering list: We found that many false positives were actually cell-line names, gene names and/or microarray names. We found that sometimes the form of a false positive was similar to that of a substitution mutation (e.g. T47D, U133A). As mentioned in Methods section, EMU uses a ‘fallible list’ of cell lines to filter cell-line names that are similar to mutation names; however, tmVar and MF do not use such a list. We included the HUGO gene list, in-house cell-line name list and other mutation-like object list in the filtering list which includes EMU’s fallible list of cell lines.
2. Non-informative mutations: tmVar and EMU find as many variants as possible, even when there is an insufficient amount of variant information to convert variants to wNm form. In many cases, variants with missing information are uninformative, ambiguous and generate false positives. Identified variants that are not in wNm form are filtered.
3. Protein substitution probability matrix filter: We used the protein substitution matrix PAM140 to filter amino acid mutations that are unlikely to occur (20).
4. Synonymous mutation: In the post-processing step, we also filtered synonymous mutations (e.g. L367L).
5. Irregular/Imbalanced variant forms including unexpected space in between variant forms: From tmVar’s results, we found that some cases have an imbalance between wild-type and mutation-type mentions. For example, from the text ‘factor V 1691G > A [PMID17003923],’ tmVar picked only ‘V 1691G’ instead of ‘1691G > A’ and translated it into SUB|V|1691|G, which produced false positives in tmVar’s results. We removed these irregular/imbalanced mutations from the text in the post-processing module.
6. Special symbols: tmVar and EMU misidentified minus (−), plus (+) and asterisk (*) symbols. For example, the minus symbol (−) in ‘Arg-23Thr’ or ‘A-23T’ denotes location. However, in ‘Arg-23-Thr’ or ‘A-23-T,’ the minus symbol (−) is not used as a minus sign but a hyphen. In the post-processing module, these special symbols were re-translated to denote the proper meaning.
7. Preposition ‘for’ processing: EMU processes all the mutation text lines and the preposition ‘for’ in the opposite way. For example, it processes ‘substitution of arginine for methionine’ as Arg ⇒ Met, which is actually Met ⇒ Arg. We fixed these problems in our post-processing module. If the preposition ‘for’ appears in between amino acids A and B, we translated it into B ⇒ A. This changed many of the false positive results to true positives.

All the above rules will increase the precision of tmVar and EMU. Rules 6 and 7 can improve both precision and recall in some cases. Table 2 shows the comparison of all the five methods that incorporated the post-processing module.

**Figure 3.**
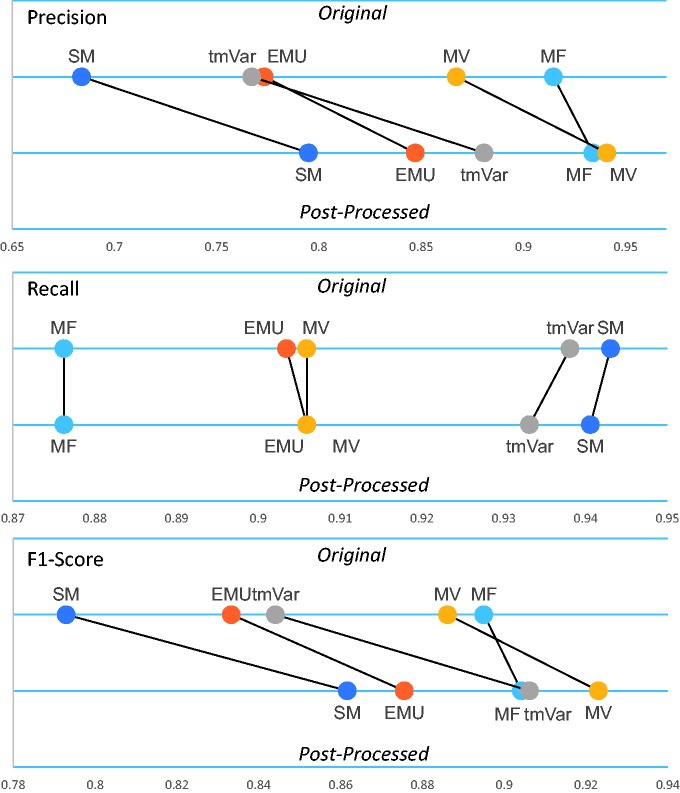
The post-processing module’s performance on the BRONCO corpus.

Table 2 clearly shows that the post-processing module improved the F1-score of all the methods. Figure 3 illustrates that the post-processing module improved the precision, recall and F1-score of the BioNLP methods used for the BRONCO experiments. (Supplementary Figure S2 illustrates the results of the rest of the corpora’s experiments.) The post-processing module improved the precision and F1-score of EMU and tmVar. MF, which extracts only simple point mutations from texts, did not improve as much as the other methods. The post-processing module also improved the precision and F1-score of the MV and SM methods.

### Comparison of running time

BioNLP methods need to be scalable and perform efficiently because they extract variants from large corpora (e.g. PubMed has > 24 million records and adds ∼3000 new abstracts per day). Thus, the running time of BioNLP methods is important. The running times of these methods are reported in Table 3.

**Table 3.** Running time of the three methods

Corpus	MF (508 abstracts)	EMU (109 abstracts)	tmVar (500 abstracts)	BRONCO (108 full-texts)
MutationFinder	28 s	7 s	31 s	139 s
EMU	75 s	17 s	77 s	201 s
tmVar	61 s	23 s	70 s	952 s

All methods were tested on a computer with an Intel Core i5-4670 3.4 GHz CPU, 24 GB RAM and Windows 10 64-bit system. As shown in Table 3, MF had the fastest running time when it was used for the abstract-based corpora and full-text corpus (BRONCO). Both EMU and tmVar had the same running time when they were used for the abstract-based corpora. However, tmVar had the slowest running time when used for BRONCO and was about 8-fold slower than MF and EMU. The average running times (per abstract) for MF, EMU and tmVar are 0.06, 0.15 and 0.14 s, respectively. As the current PubMed collection has around 24 million abstracts, the estimated total running times will be 394, 1009 and 919 machine hours for MF, EMU and tmVar, respectively. Because this task is parallelizable, all the methods are scalable.

## Discussion

### Lessons learned

We analysed the three BioNLP methods and the two variant extraction ensemble approaches. We believe that there is room for improvement. Although the previously described post-processing module correctly identified many of the variants that were misidentified by each of the BioNLP methods, some of the variants described in the text are false positives (due to typos by authors). We qualitatively evaluate the following examples and provide the lessons learned from our analysis.

Example 1:

‘In a patient, a C-to-G transition was identified at nucleotide 857 in exon 8 that resulted in a substitution of alanine for proline at amino acid 286 in the first calcium binding EGF domain’. [PMID21080147, obtained from the tmVar data set]

Answer provided by the original source: none

Correct Answer (updated in the unified format): P|286|A

EMU: A|286|P

tmVar: |SUB|P|286|A

MF: P|286|A

Example 1 was obtained from the tmVar corpus; however, the underlined variant was excluded from the tmVar answer set. We updated the variant information in the new tmVar answer set that we provide so that it includes the correct variant which is P|286|A (wild type: proline; position: 286; mutant: alanine). All three BioNLP methods identified and retrieved this variant from the text. However, only tmVar and MF correctly retrieved this variant. EMU incorrectly retrieved this variant. When finding variant mentions such as ‘alanine for proline’ in the text, EMU finds information of the position (which is 286 in this example) near the variant. EMU extracts variants in natural language form simply taking the first amino acid (alanine) as the wild type and the second amino acid (proline) as the mutant. We found that EMU incorrectly extracts many examples in this step. We rectify this issue using our post-processing module.

Example 2:

‘Fifteen patients were heterozygous for the G–≥A polymorphism at nucleotide 5557, which causes substitution of asparagine for aspartic acid at position 1853 of the ATM protein’. [PMID17517479, obtained from the EMU data set]

Answer provided by the original source: AS*N* | 1853|ASP

EMU: G|1853|A, AS*N* | 1853|ASP

tmVar: |SUB|G|5557|A, p|SUB|D|1853|N

MF: *N* | 1853|D, D|1853|N

Correct answer (updated in the unified format): ASP|1853|ASN (OR D|1853|N), G|5557|A

In Example 2, we found that the answer provided by the source is wrong. The correct answer is p.D1853N (ASP|1853|ASN) or c.G5557A (coding DNA location is 5557). tmVar is the only method that correctly extracted both variants at DNA and protein levels. EMU extracted the wrong coding DNA location and reversed the order of amino acids. These issues can be resolved using the post-processing module we provide. MF notated the extracted variant twice: one notation was correct and the other was incorrect.

Example 3:

‘The Turkish patient and her affected relatives all had a heterozygous A to G transition at codon 557 (AAG–≥GAG) of exon 10 of MEN1 that results in a replacement of lysine by glutamic acid’. [PMID16840830, obtained from the tmVar data set]

Answer provided by the original source: |SUB|AAG||GAG

Correct answer (updated in the unified format): K|557|E

EMU: K|557|E

tmVar: |SUB|AAG||GAG

MutationFinder: none

In Example 3, we can see the results returned by each of the methods. Notice that 557 is the location of the codon, and not the location of the coding nucleotide. Both EMU and tmVar did not retrieve this as A557G but they skipped this part and found AAG–>GAG which is the right answer. In this case, A557G is the wrong answer because 557 is the location of an amino acid and not of a nucleotide. tmVar found this as AAG–>GAG, but is still missing information on the location. EMU translated AAG and GAG into K (lysine) and E (glutamic acid), respectively, and matched them with the location (557) and thus returned the best result.

Another lesson that we learned from this study is the use of proper nomenclature in describing variants. Standard nomenclature for variants described in the literature could increase the efficiency of variant extraction by BioNLP. Some of the variant forms described in the text were not recognized by BioNLP or human annotators/readers. We strongly encourage authors to follow HGVS (11) mutation nomenclature when describing variants in publications.

### Assessing overlapping variants extracted using BioNLP methods

**Figure 4.**
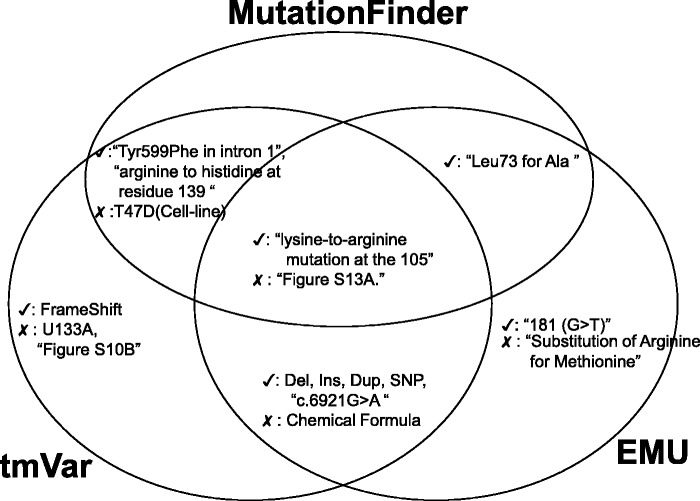
Examples of true and false positives identified by the three methods.

Next, we assess the true positives and false positives identified by the BioNLP methods and we use a Venn diagram to illustrate the common true and false positives (Figure 4). All the three methods could extract mutations in wNm form successfully. However, in some cases, these algorithms misidentified names (e.g. U133A, T47D, Supplementary Figure S10A) in the text. For example, although U133A is the name of the Affymetrix microarray platform, MF and tmVar mistakenly identified it as a variant. Also, MF and tmVar mistakenly extracted T47D, which is the name of a cell line. According to our evaluation, EMU could eliminate these false positives as it uses a fallible list for filtering these mutation-like entities, especially cell lines. Interestingly, all three methods misidentified supplementary figure information as variants (e.g. Supplementary Figure S13A). The post-processing module can filter these false positives.

Overall, tmVar had the best recall as it can extract variants in various forms. It is the only BioNLP method that can retrieve frameshift mutations. Conversely, as MF can retrieve only point mutations, it had the lowest recall. However, if the text contains only point mutations, MF can correctly identify these variants and achieve high precision.

Extracting the exact location of a variant in a text often creates false positives. As previously mentioned, the numbers in a variant form notate either the coding DNA location or the protein location; however, the BioNLP methods are not optimal for determining the type of location information of a variant. Future work in determining the type of location can help improve the precision of the BioNLP methods.

### Mutations in biological context

As shown in the Results section, among the four corpora, both tmVar and EMU achieved good performance in extracting mutations. Specifically, tmVar achieved the best coverage in extracting different kinds of mutations (e.g. SNPs, indels). On the other hand, EMU performed the best in changing codons to amino acids, finding the exact location numbers of variants and identifying related gene names and validation filters using online databases such as RefSeq. Combining the regular expressions of EMU and tmVar, and integrating the use of CRFs can improve the performance of variant extraction methods. We strongly believe that papers that follow the HGVS nomenclature will make it easier for BioNLP methods to extract mutations from the literature.

The mutation extraction task represents the first step in applying BioNLP methods that assist in the functional annotation and interpretation of variants. Extracting only variants but not their biological context is useless. We believe that future variant extraction methods should be able to extract variants and their biological context from text. For example, a variant (e.g. V600E) found in a gene (e.g. BRAF) that is correlated with its biological context such as drug sensitivity (e.g. sensitive to vemurafenib) or disease phenotype (e.g. melanoma) is more meaningful than just the identified variant itself. Biological context is crucial for building knowledge bases of clinically relevant variants. As exemplified by the manually curated knowledge bases such as My Cancer Genome (8) or MD Anderson PCT (https://pct.mdanderson.org), mutation information such as phenotypes, other biomedical entities and relationships with drugs should be annotated.

### Gene mapping evaluation of EMU

Among the three BioNLP methods that we compared in this study, EMU is the only method that attempts to extract variant-gene relationships from the literature. Currently, EMU uses co-occurrence information and amino-acid sequence verification for finding related genes.

We evaluated EMU’s gene mapping module using BRONCO. EMU consists of two different modules: the first module retrieves mutations and genes from articles, and the second module verifies the relations between the mutations and genes the first module retrieved. Instead of finding genes related to a variant, the first module simply lists all the genes that appeared in the same article. The second module, which is called the SEQ_filter, finds genes most related to each variant using RefSeq data. This process requires an online connection and a long running time. Table 4 shows the statistics of EMU’s gene mapping evaluation. The overall precision and recall of EMU’s gene mapping that used BRONCO’s variant-gene relations were 0.287 and 0.604, respectively. Many false positive cases were due to poor NER processes. Only 69.37% of the gene names retrieved from the literature using EMU’s NER process was correct. Also, we evaluated only the cases where NER is correctly performed. In these cases, precision and recall are 0.443 and 0.832, respectively. As the result shows, EMU’s gene mapping method is not accurate enough to replace manual curation, and EMU’s variant-gene tagging still requires improvement. We found that using only co-occurring information results in poor accuracy, and that online sequence filtering is time consuming.

**Table 4.** The result of EMU’s gene mapping module that used the BRONCO data set

	All variants	Only protein variants	NER was successful
Number of variants	333	308	232
Gene numbers before filtering	4467	4416	3450
Gene numbers after filtering	681	647	524
Precision	0.283	0.287481	0.443
Recall	0.580	0.604	0.832

In a recent study, Burger *et al.* (21) suggested utilizing a crowdsourcing approach to resolve the variant-gene mapping problem. They used EMU to extract variants and their related gene candidates from 810 abstracts, and hired Amazon Mechanical Turk workers to find the relations. Although their crowdsourcing approach improved the gene-variant mapping results, it is expensive and not scalable.

### Related work

Yepes *et al.* (15) compared the performance of several BioNLP methods including tmVar, EMU, MF and other MF-based methods (SETH and OMM), and found that tmVar and EMU achieved good performance in variant extraction. However, this comparison is limited due to the lack of resources. The Variome corpus contains only 10 texts, and COSMIC and InSiGHT were used for evaluating only recall. tmVar, EMU and MF provide their own gold-standard data sets for evaluation; however, these data sets are in different formats and the data sets do not accurately represent biomedical literature. For example, the EMU corpus contains only prostate cancer and breast cancer-related text. While the MF corpus does not contain any non-substitution mutations, the tmVar corpus contains many non-substitution mutations, which is unrealistic. The MF data set is outdated and thus cannot completely represent biomedical literature or achieve representativeness. For these reasons, Yepes *et al.* could not use the data set of each tool for the comparison of the tools. They reported the results of the comparison, which were obtained by the author of each tool. However, for this study, we standardized the output from public corpora, which allowed us to cross-validate tmVar, EMU and MF. Based on our study, we found that both tmVar and EMU achieved good performance in extracting variants.

### Future work using BRONCO

To assist the community in identifying variants with their biological context, we have annotated BRONCO that contains mutation-gene and mutation-phenotype relationships from 108 full-text papers. All of these relationships were manually curated and verified by at least two curators; additional curators were used if needed. The variants curated in BRONCO are enriched with substitution mutations. As part of our future work, we plan to expand BRONCO with other forms of variants. BRONCO is freely available to the community, and we believe that it is a valuable resource for developing future BioNLP methods.

As we described, BRONCO contains rich variant-related annotations such as genes, diseases, drugs and cell lines. Many BioNLP systems focus on retrieving only variant mentions; however, more advanced research will focus on retrieving not only variants but also other related entities. BRONCO, a great resource, can be used as a gold-standard data set for the automatic functional annotation of variants.

## Conclusion

In summary, we developed BRONCO—a novel corpus that consists of 108 full-text papers—for objectively evaluating the performance of three BioNLP systems. We also proposed a post-processing module of the systems’ results. Using available corpora including BRONCO, we have compared the three BioNLP systems’ performance in extracting variants from scientific literature for functional annotation and interpretation. We provided ‘lessons learned’ from this work, and presented future work in developing BioNLP systems for extracting, annotating and interpreting variants. We believe that BRONCO will be a valuable resource for evaluating and training new methods in extracting biomedical entity relations from full-text publications, and prove to be an asset to the biomedical text mining research community.

## Supplementary data

Supplementary data are available at *Database* Online.
